# Social perspective for well-being and health behavior change

**DOI:** 10.3389/fpubh.2026.1989631

**Published:** 2026-10-28

**Authors:** Hatem H. Alsaqqa

**Affiliations:** 1 Faculty of Public Health and Deanship of Scientific Research, Al-Quds University, Jerusalem, Palestine; 2 Palestinian Ministry of Health, Gaza, Palestine

**Keywords:** behavior change, health promotion, public health, social theory, well-being

## Abstract

Health behavior is shaped not only by individual capabilities and intentions but also by social relationships, cultural meanings, everyday practices, and the structural distribution of resources and power. Building on practice theory, social network research, social epidemiology, social-determination-of-health traditions, and systems approaches, this Perspective develops an integrative social-systems account of health behavior change. It does not claim that these traditions are new; rather, it specifies how their complementary mechanisms interact and feed back across contexts. Structural conditions shape opportunities and constraints; networks organize exposure to resources and norms; social connection and social capital influence the support and collective capacity available through relationships; practices organize repeated activity; and culture and social identity shape what behaviors mean and whether they are socially acceptable. These processes are also affected by contradictions, unequal power, and collective action. Examples from physical activity, healthy eating, and smoking illustrate how the framework can guide multi-level health promotion. The perspective encourages researchers and practitioners to move beyond treating social influences as additional individual-level predictors and instead examine how social, cultural, institutional, and material processes jointly make health practices feasible, meaningful, and sustainable.

## 1 Introduction

Persuading people to alter their behavior in an attempt to lessen their likelihood of developing certain “lifestyle” diseases has been a major focus of public health research and practice over the past few decades. In fact, this does not appear to be an improper response because the etiology of the NCD epidemics is somewhat based in the specifics of daily life and what individuals do. Routines, habits, and accepted ways of living are more difficult to change than distal behaviors.

An overabundance of arguments, techniques, and instruments, as well as numerous tests and assessments, have been produced in response to the difficulty of changing behavior. Despite significant differences, the majority of behavior change models assume that people can make “better” decisions for themselves based on the information they are given and that these decisions that have an impact on their well-being. This interpretation has been critiqued by Ioannou (1), Thompson and Kumar (2), and most recently by nudge theory, it serves as a reminder that people are only partially logical information assessors and frequently react instinctively to their immediate surroundings (3).

However, there is currently conflicting information about the potential impact of a nudge on changing behavior related to health (4). Cohn (5) draws attention to this paradox by advocating for a theoretical reorientation within the field as well as an analytical and practical shift from health behaviors to health practices. He argues that structural or cultural perspectives are not always going to oppose the underlying primacy of the individual and their decisions.

Researchers draw attention to the potential and usefulness of an alternative social theoretical tradition that sees daily life patterns (and their effects on health) as products of the synchronization and the integration of social practices that endure over time and space and they are replicated and altered by those who “carry” them. Researchers argue that public health policy would be better served by concentrating on the “lives” of social behaviors, treating them as independent subjects of investigation and action (6).

However, theory-based approaches have generally played a significant role in the creation of effective health behavior promotion (7). Regular exercise, a balanced diet, adequate sleep, abstaining from recreational drugs and smoking, and restricting or giving up alcohol are all examples of a healthy lifestyle. These problems gained attention during the global Covid-19 pandemic, when businesses lost approximately $154 billion as a result of loneliness-related absenteeism (8).

Although the social cognitive paradigm’s attitude-behavior and capability-behavior models were the main focus of early health behavior theory research (9), more recent methods have expanded to include dual-process approaches (10), action control models (11, 12), needs models, and natural environments (13). Despite this theoretical development, there is still a lack of coherence in the theoretical framework surrounding the various social processes associated with health behaviors.

Moreover, social constructions are rarely the main emphasis of health behavior theories, even though they almost mention social elements in health behavior (14). Similarly, at the individual-level, social factors (e.g., perceived social support, relatedness needs, and perceived role modeling) are generally provided with less comprehensiveness compared to psychological constructs such as self-efficacy (or perceived behavioral control), goals, and intentions (9).

Furthermore, those who are thought to be the most deprived have a life expectancy that is more than 18 years less than that of those who are thought to be the least deprived (15). The social determinants at work are numerous and include, but are not limited to, social inclusion and nondiscrimination, housing quality, transportation and neighborhood safety, physical activity, health services, air and water quality, and economic stability (access to high-quality education and employment) (16).

Although the public underestimates the influence of social connection on physical health and lifespan, it is well acknowledged that social connection has a considerable impact on emotional well-being (17). Increased social connectivity is associated with lower risk, according to strong interdisciplinary scientific evidence, whereas social negativity and social isolation raise the risk of morbidity and mortality from a range of infectious and chronic diseases and disorders (18). Many health behavior interventions lack social connection in one or more ways, despite the advantages of social perspectives, which may greatly exacerbate public health issues.

Nonetheless, the theories reviewed in this article are complementary rather than competing explanations of health behavior. Practice theory explains how behaviors become organized as routine practices through meanings, materials, and competences. Social network and social connection perspectives explain how relationships provide resources, information, support, and normative influence. Homophily helps explain why social influence is patterned by similarity and group composition, while social identity explains why particular norms and behaviors become meaningful to members of social groups. Structural approaches place these processes within wider systems of power, policy, institutions, and unequal access to resources. Taken together, these perspectives suggest that behavior change is produced through interactions between structure, relationships, practices, identity, and individual action.

This integration also changes how interventions should be designed. Individual-level education or motivation strategies may be necessary, but their effects can be constrained when healthy practices are materially difficult, socially unsupported, or inconsistent with dominant norms and identities. Conversely, structural or community interventions may create opportunities for change without automatically changing everyday practices. A more useful approach is therefore to combine interventions across levels and examine how they interact. Systems thinking and implementation science are particularly relevant because they can help identify interactions, adaptations, feasibility, and sustainability in real-world settings (19, 20).

Finally, I argue that the main limitation of many health behavior approaches is not the absence of social variables, but the tendency to treat social influences as additional predictors of individual behavior. My perspective instead views behavior change as a dynamic process across interconnected levels. Structural conditions determine resources and opportunities; social networks and norms shape what is expected, supported, or discouraged; everyday practices organize how behaviors are repeatedly performed; and social identities influence whether behaviors become meaningful and acceptable. These processes are reciprocal: people can reproduce existing arrangements or collectively modify them.

These arguments build on, rather than replace, established systems-oriented and relational accounts of health. Practice theory has already challenged the reduction of everyday activity to individual choice, while social epidemiology and Latin American social medicine and social-determination-of-health traditions have emphasized how social relations, political economy, and unequal distributions of power become embodied in health. Ecosocial and dialectical approaches similarly examine reciprocal relations between people, institutions, history, and environments (19–24). The contribution offered here is therefore integrative: it brings these traditions into an explicit explanatory architecture that distinguishes their mechanisms while showing how they interact, reproduce inequality, and can be changed through collective and institutional action.

## 2 Structural conditions of social life

Structural perspectives make a distinctive contribution to the proposed framework by specifying the conditions that distribute opportunities, constraints, resources and power before individuals encounter particular social environments. Socioeconomic position, employment, education, housing, neighborhood conditions, institutional arrangements and public policy do not simply add background risk; they shape the material and social circumstances in which relationships are formed and everyday practices become possible or difficult (21, 25–27). This contribution therefore establishes the outer conditions of the proposed social system, while leaving open the question of how those conditions become behaviorally consequential.

However, the key analytical implication is that structural conditions should not be treated as distal, unidirectional determinants. Their effects can operate through the organization of social opportunities, network composition, access to supportive relationships, available resources and the practices that can be sustained. For example, unequal housing, employment or neighborhood conditions may influence who interacts with whom, what forms of participation are feasible, and which resources are accessible through social relationships. These pathways connect structural explanations directly to the relational and practice-based perspectives (21, 25, 26, 28).

Giddens’ structuration perspective adds a complementary mechanism: structures constrain and enable action but are also reproduced or transformed through action (29). In the integrated framework (Figure 1), this means that structure is both a condition of behavior and an outcome that can be modified through repeated individual and collective practices. This reciprocal interpretation differentiates the proposed framework from models in which structural determinants simply sit at the top of a hierarchy of causes.

**Figure 1:**
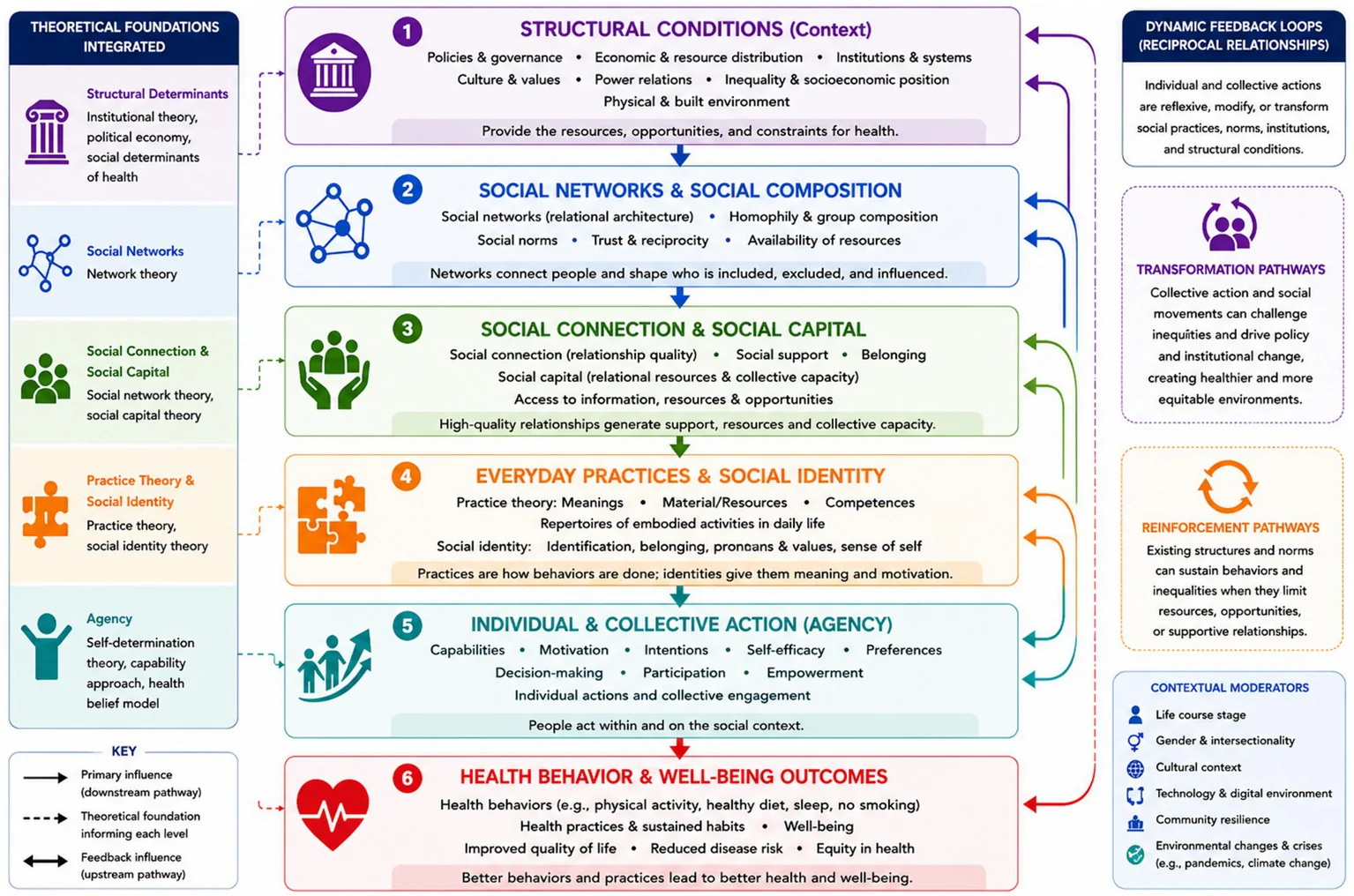
Social-systems framework for health behavior change and well-being.

The distinctive contribution of structural theory is therefore not to explain the immediate mechanism of behavior change, but to explain why the social and material opportunities through which other mechanisms operate are distributed unequally. This becomes particularly important for equity: interventions that alter behavior without altering unequal access to resources, institutions or supportive environments may produce limited or uneven effects (21, 22, 27, 30, 31).

## 3 Social networks and architecture of behavior

Social network perspectives contribute a different level of explanation by specifying how structural conditions are translated into patterned relationships among people and organizations. Network analysis focuses on the structure, position and characteristics of ties, including network size, density, centrality, reciprocity and composition (32–35). Within the proposed framework, networks therefore function as a relational architecture connecting wider social conditions with individual experience.

However, the analytical value of network theory is that it prevents social influence from being reduced to a generic “social factor.” Network position can shape exposure to information, resources, norms and support, while network structure can affect how rapidly or widely behaviors and expectations circulate (32, 35–38). This perspective also makes visible mechanisms that individual-level models may overlook, including diffusion, clustering, selection and the role of particular actors or ties in sustaining change.

Homophily should be understood within this network architecture. It contributes specifically by explaining why networks are often socially and behaviorally similar: people may form or maintain relationships with others who share characteristics, experiences or social positions (35, 39). Importantly, observed similarity cannot automatically be interpreted as social influence. It may arise through selection into similar networks, shared environments or subsequent influence. Distinguishing these mechanisms is essential when interpreting network effects on health behavior (39, 40).

Nonetheless, this distinction strengthens the integrated framework because it links network processes back to structural conditions. Residential segregation, socioeconomic inequality, workplace organization and access to institutions can shape opportunities for interaction, while homophily can reinforce similarity once networks are formed. Networks therefore both reflect social structure and mediate its consequences. The distinctive contribution of network theory is consequently to explain the relational pathways through which opportunities, norms and resources reach individuals, while homophily helps explain how these pathways become patterned across groups.

## 4 Social connection and social capital

Social connection and social capital extend the network perspective by asking not only who is connected to whom, but what relationships provide and how those resources influence health. Social connection encompasses structural, functional and qualitative dimensions of relationships, ranging from the availability of social ties to the support and positive quality experienced within them (41, 42). Its distinctive contribution is therefore the functional and experiential dimension of relational life: network structure may create opportunities for contact, but connection determines whether those relationships are meaningful and supportive.

This distinction is important because the existence of a network does not guarantee beneficial social effects. Relationships can provide emotional and practical support, information and encouragement, but they can also transmit stress, unhealthy norms or exclusion (43, 44). Social connection consequently represents a mechanism through which network structure becomes consequential for everyday behavior and well-being rather than an additional independent predictor.

However, social capital adds a further, related but distinct contribution by emphasizing resources embedded in relationships and the collective capacity generated through trust, reciprocity, shared norms and community organization (45–53). Whereas social connection emphasizes the presence, function and quality of relationships, social capital focuses more explicitly on what individuals and communities can access or accomplish through those relationships. The concepts therefore overlap but should not be treated as interchangeable.

Within the integrated framework, social connection and social capital occupy the space between network architecture and everyday action. Structural conditions influence access to relationships and the distribution of relational resources; networks determine pathways through which those resources circulate; social connection determines whether relationships are supportive and meaningful; and social capital captures the resources and collective capacities that can be mobilized through them. Their integration therefore helps explain why similar network structures can produce different behavioral consequences across contexts.

## 5 Everyday practices and social identity

Practice theory makes a distinctive contribution by explaining how social and structural conditions become incorporated into repeated everyday activity. Rather than treating behavior as a sequence of individual choices, practice approaches conceptualize activities as organized through the interaction of meanings, materials and competences (54–56). This provides a mechanism connecting social environments to behavioral repetition: structures influence the material and institutional conditions of practices, while repeated performance helps reproduce routines and the settings in which they occur.

However, this perspective complements, rather than duplicates, social network theory. Networks explain relational exposure and resource flows; practice theory explains how those social and material conditions are assembled into routinized activity. For example, social support may make physical activity more socially acceptable, but participation also depends on time, transport, facilities, skills and meanings attached to being active. Practice theory therefore identifies the conditions under which social influence becomes a stable pattern of everyday behavior (57, 58).

Moreover, social identity contributes a different mechanism by explaining how group membership, belonging and shared meanings influence whether behaviors are perceived as appropriate, desirable or identity-consistent (59–63). Identity is particularly important because people do not respond to norms and practices only as isolated individuals; they interpret them in relation to valued social groups. A behavior can therefore become more sustainable when it is incorporated into a valued identity, while practices can also strengthen identification when they are repeatedly shared with others.

Nevertheless, the analytical distinction between practice and identity is consequently useful. Practice theory explains the organization and reproduction of activity, whereas social identity explains the social meaning and group-based significance attached to that activity. Their integration addresses a limitation of each perspective considered alone: practices cannot be fully understood without considering why some meanings and forms of participation become valued, while identity cannot by itself explain the material and practical conditions required to enact identity-consistent behavior.

## 6 Culture, meaning and embodiment

Culture is not simply a background characteristic or a synonym for social norms. It comprises shared and contested meanings, values, symbols, histories, and practical understandings through which people interpret bodies, health, risk, responsibility, and appropriate ways of living. Cultural themes can influence what is regarded as normal, desirable, respectable, or feasible, while also varying within groups and changing over time. Work on practice and symbolic interaction emphasizes that meanings are reproduced through everyday activity rather than existing only as abstract beliefs (56, 59, 64).

Within the proposed social-systems perspective, culture interacts with material conditions, networks, practices, and identity. For example, food choices are shaped not only by knowledge or motivation but also by culturally meaningful meals, family routines, affordability, availability, and expectations about hospitality and body size. Comparative research on healthy eating illustrates how cultural orientations can shape dietary practices, while research on social norm interventions cautions that interventions may fail when they overlook the meanings and relationships through which norms are sustained (49, 50). Studies of mask-wearing also show how sociocultural context can shape the uptake of health-protective practices (51). Culture therefore mediates how structural change is interpreted and embodied; it should be examined as a dynamic process, not used to stereotype populations or treat cultures as internally uniform.

This perspective also highlights embodiment: social and material conditions are lived through bodies, routines, stress, access to food and activity, and exposure to risk. Cultural meanings may support health-promoting change, but they can also stigmatize people or normalize practices that are harmful. Intervention design should therefore engage with local meanings and community knowledge while also addressing the structural conditions that limit choice. Participatory approaches can help identify which meanings matter, whose interpretations dominate, and how healthier practices can become socially legitimate without blaming individuals or communities.

## 7 What the integration adds: reciprocal dynamics across the social system

The central contribution of the proposed framework is therefore not the accumulation of additional social determinants, but the specification of relationships among distinct mechanisms. Structural conditions shape opportunities for interaction and the distribution of resources; networks organize exposure to people, norms and information; homophily helps explain network composition; social connection and social capital determine the quality and resources available through relationships; practices organize repeated activity; and social identities give behaviors social meaning. These processes are interconnected rather than sequential.

However, the framework is explicitly reciprocal. Structural conditions can shape network formation, but networks and collective action can also reproduce or challenge institutional arrangements. Social relationships can support healthy practices, while repeated participation in shared practices can strengthen relationships and identities. Identities influence behavioral expectations, while collective practices can transform what becomes normative or valued within a group. Individual action therefore occurs within socially patterned opportunities and constraints, but individuals and groups also have the capacity to modify those conditions (21, 29, 31).

This integration also clarifies where perspectives overlap and where they remain analytically distinct. Networks and social capital both concern relationships, but network theory primarily addresses relational structure whereas social capital emphasizes resources and collective capacity. Social connection overlaps with both but focuses more directly on the existence, function and quality of meaningful relationships. Practice theory and social identity both address meaning, yet practice theory emphasizes the organization of activity while identity emphasizes belonging and group-based self-understanding. Structural perspectives encompass the wider distribution of power and resources within which all of these processes operate. These distinctions allow the framework to integrate the theories without collapsing them into a single undifferentiated construct.

Nonetheless, the integration consequently changes the causal question from “which social factor predicts behavior?” to “through which interacting mechanisms do social and structural conditions make particular behaviors feasible, supported, meaningful and repeatable, and how can action alter these conditions?” This is important for interpreting empirical associations. For example, an association between social connection and physical activity could reflect network selection, social influence, access to resources, shared environments, identity processes or combinations of these mechanisms (36, 39, 40). The integrated framework encourages these mechanisms to be examined mutually rather than inferred from a single association.

## 8 Social contradictions, power and collective action

Social systems are not harmonious or uniformly beneficial. They contain contradictions between commercial interests and public health, between institutional priorities and community needs, and between individual responsibility narratives and the unequal conditions that shape what people can do. These tensions influence which health problems receive attention, whose knowledge is recognized, how resources are allocated, and which practices are supported or constrained. Structural and ecosocial approaches emphasize that power and political-economic arrangements are integral to the production of health inequalities, not merely contextual variables (21–25, 30).

Contradictions can also generate resistance, negotiation, and collective action. Community organizing and social movements may challenge inequitable environments, demand safer public spaces, improve access to services, or contest policies that shift responsibility onto individuals without changing the conditions of daily life. Collective action can create new networks and shared identities, but it may also encounter institutional resistance, unequal participation, or conflicts within communities. Research on community organizing and collective behavior helps show why social change cannot be understood solely as the accumulation of individual choices (31, 65, 66).

An analytical focus on contradiction does not assume that all conflict is progressive or that collective action always produces equitable outcomes. Rather, it asks who has decision-making power, whose interests are served, which groups bear the costs of change, and how institutions respond to challenge. For health promotion, this means examining power and resource distribution alongside social support, norms, practices, and identity. Interventions should create routes for meaningful community influence and monitor whether they reduce or reproduce exclusion.

## 9 Implications for health promotion and intervention design

The analytical value of the framework is ultimately reflected in how it changes intervention design. If behavior is produced through interacting mechanisms, interventions directed only at individual motivation may have limited effects when healthy practices are materially difficult, socially unsupported or inconsistent with valued identities. Conversely, structural or environmental interventions may create opportunities for change without ensuring that those opportunities are incorporated into everyday practices or supported by social networks.

Therefore, the framework suggests that intervention points should be selected according to the mechanism that needs to change. Structural interventions can alter resources, rules, environments and power relations; network interventions can modify connectivity, diffusion and peer influence; social-connection interventions can strengthen meaningful support; social-capital approaches can mobilize collective resources; practice-oriented interventions can change materials, competences and meanings surrounding routine activities; and social-identity approaches can strengthen identities that make healthy behavior socially meaningful. The contribution of the integrated framework is to show how these intervention points can be coordinated rather than treated as unrelated strategies (19–22, 27, 30, 31, 37, 38).

Nevertheless, this also has implications for implementation and equity. The same intervention mechanism may operate differently across contexts because structural conditions determine access to resources, network opportunities and feasible practices. Multi-level interventions should therefore be evaluated not only by whether they change behavior, but by how mechanisms interact, for whom they work, and whether they redistribute opportunities and resources equitably. Systems-thinking and implementation approaches are particularly useful for examining these interactions, adaptations and feedback processes in real-world settings (19, 20).

## 10 Applying the social-systems perspective to specific health practices

Physical activity illustrates why no single level of intervention is sufficient. Individual motivation and confidence may support participation, but active travel and recreation also depend on safe streets, transport, time, facilities, cost, workplace and family routines, and whether activity is socially supported. Networks can provide companionship and encouragement, while practice-oriented approaches attend to the materials, skills, and meanings that make activity repeatable. Evidence on rural young people, healthy eating determinants, and social support for physical activity illustrates the relevance of combining individual, relational, and environmental explanations (43, 57, 58). A coordinated response might therefore combine accessible facilities and safe routes with community-led groups and opportunities to integrate activity into daily routines.

Healthy eating similarly emerges from the interaction of food availability and affordability, household resources, cultural meanings, social expectations, skills, and routines. Education about nutrition may have limited effect where healthy foods are unaffordable or unavailable, while changing retail or institutional food environments may not be sufficient if new options conflict with established practices or social meanings. The framework directs attention to how material access, cultural themes, family and peer networks, and practical competences jointly shape food practices (49, 58). Interventions can combine access measures, supportive institutional food policies, culturally responsive community work, and practical opportunities to develop skills.

Smoking provides a third example. Smoking practices may be sustained by peer networks, shared routines, stress, commercial influences, and local social norms, while cessation can be constrained by socioeconomic disadvantage and unequal access to support. Research on neighborhood context and smoking among young people underscores that behavior must be understood in relation to social environments, rather than solely as a matter of individual knowledge or will (67). Norm-focused interventions may help, but they need to distinguish between correcting misperceptions and changing the social conditions that reproduce smoking practices (50). A multi-level response may combine accessible cessation support with smoke-free settings, action on commercial and environmental influences, and community engagement.

Across all three examples, the framework is used as a set of linked questions rather than a checklist of independent determinants: what structural conditions enable or constrain the practice; how are relationships and resources organized; which cultural meanings and identities shape acceptability; how is the practice reproduced in everyday life; and where can policy, community action, or service delivery alter these interacting mechanisms? Evaluation should examine not only behavioral outcomes but also reach, equity, adaptation, unintended effects, and sustainability. The examples are illustrative applications of the perspective, not claims that a single intervention package will work uniformly across contexts.

## 11 Conclusion

The central contribution of the proposed framework is to reposition health behavior change as a dynamic social-systems process rather than primarily an individual decision. The structural perspectives explain the distribution of opportunities, resources and power; network theory explains relational architecture and exposure; homophily explains patterned network composition; social connection and social capital explain the quality and resources of relationships; practice theory explains how behaviors become organized and reproduced in everyday routines; and social identity explains how behaviors acquire meaning through group membership and belonging. Their integration adds explanatory value by showing how these mechanisms interact and feedback on one another.

For public health practice, the implication is not to abandon individual-level approaches, but to embed them within interventions that also modify social environments, everyday practices, institutional conditions, and power relations. For research, the priority is to test these interactions using systems and implementation approaches and to identify mechanisms that are transferable across contexts while remaining sensitive to local culture and inequality. However, a social perspective becomes most useful when it guides the design, implementation, and evaluation of interventions that make healthy practices socially supported, materially feasible, and equitably accessible.

Nevertheless, future research should move from describing social influences to testing how they interact and how these interactions can be changed. Systems-thinking and complexity-informed methods can be used to map feedback loops among social networks, practices, institutions, and health outcomes. Implementation science can help identify which multi-level strategies are feasible, acceptable, adaptable, and sustainable. Participatory and interdisciplinary research should involve communities, practitioners, policymakers, sociologists, behavioral scientists, and health-system researchers in designing interventions. Digital public health provides opportunities to strengthen social connection and peer support, but research should examine both benefits and potential harms, including exclusion and unequal access. Finally, equity should be treated as a core outcome: interventions should examine who benefits, who is excluded, and how policies and implementation strategies may redistribute resources, opportunities, and power.

## Data Availability

The original contributions presented in the study are included in the article/supplementary material, further inquiries can be directed to the corresponding author.
